# The impact of COVID-19 on the diagnosis and treatment of HCC: analysis of a nationwide registry for advanced liver diseases (REAL)

**DOI:** 10.1038/s41598-024-53199-6

**Published:** 2024-02-03

**Authors:** Kazuya Okushin, Ryosuke Tateishi, Shinya Hirakawa, Hisateru Tachimori, Koji Uchino, Ryo Nakagomi, Tomoharu Yamada, Takuma Nakatsuka, Tatsuya Minami, Masaya Sato, Mitsuhiro Fujishiro, Kiyoshi Hasegawa, Yuichiro Eguchi, Tatsuya Kanto, Hitoshi Yoshiji, Namiki Izumi, Masatoshi Kudo, Kazuhiko Koike

**Affiliations:** 1 Department of Gastroenterology, Graduate School of Medicine, The University of Tokyo, 7-3-1 Hongo, Bunkyo-ku, Tokyo, 113-8655 Japan; 2 Department of Infection Control and Prevention, Graduate School of Medicine, The University of Tokyo, Tokyo, Japan; 3 Department of Healthcare Quality Assessment, Graduate School of Medicine, The University of Tokyo, Tokyo, Japan; 4 Hepato-Biliary-Pancreatic Surgery Division, Artificial Organ and Transplantation Division, Department of Surgery, Graduate School of Medicine, The University of Tokyo, Tokyo, Japan; 5 Endowed Course for Health System Innovation, Keio University School of Medicine, Tokyo, Japan; 6 Liver Center, Saga University Hospital, Saga University, Saga, Japan; 7 The Research Center for Hepatitis and Immunology, National Center for Global Health and Medicine, Chiba, Japan; 8 Department of Gastroenterology, Nara Medical University, Kashihara, Nara Japan; 9 Department of Gastroenterology and Hepatology, Musashino Red Cross Hospital, Musashino, Japan; 10 Department of Gastroenterology and Hepatology, Kindai University Faculty of Medicine, Osaka-Sayama, Japan; 11 Kanto Central Hospital, Tokyo, Japan

**Keywords:** Hepatology, Epidemiology

## Abstract

The number of cancer cases diagnosed during the coronavirus disease 2019 (COVID-19) pandemic has decreased. This study investigated the impact of the pandemic on the clinical practice of hepatocellular carcinoma (HCC) using a novel nationwide REgistry for Advanced Liver diseases (REAL) in Japan. We retrieved data of patients initially diagnosed with HCC between January 2018 and December 2021. We adopted tumor size as the primary outcome measure and compared it between the pre-COVID-19 (2018 and 2019) and COVID-19 eras (2020 and 2021). We analyzed 13,777 patients initially diagnosed with HCC (8074 in the pre-COVID-19 era and 5703 in the COVID-19 era). The size of the maximal intrahepatic tumor did not change between the two periods (mean [SD] = 4.3 [3.6] cm and 4.4 [3.6] cm), whereas the proportion of patients with a single tumor increased slightly from 72.0 to 74.3%. HCC was diagnosed at a similar Barcelona Clinic Liver Cancer stage. However, the proportion of patients treated with systemic therapy has increased from 5.4 to 8.9%. The proportion of patients with a non-viral etiology significantly increased from 55.3 to 60.4%. Although the tumor size was significantly different among the etiologies, the subgroup analysis showed that the tumor size did not change after stratification by etiology. In conclusion, the characteristics of initially diagnosed HCC remained unchanged during the COVID-19 pandemic in Japan, regardless of differences in etiology. A robust surveillance system should be established particularly for non-B, non-C etiology to detect HCC in earlier stages.

## Introduction

Primary liver cancer (PLC) is the third leading cause of cancer-related mortality worldwide, accounting for 8.3% of all cancer-related deaths^1^. Hepatocellular carcinoma (HCC), the most common type of PLC, accounts for 80% of all PLC cases^2^. The high-risk population for HCC is limited to those with chronic liver disease, particularly cirrhosis, with hepatitis B, alcohol consumption, and hepatitis C contributing to 33%, 30%, and 21% of liver cancer deaths, respectively^3^. The fact that this high-risk group is limited to a small population has contributed to the establishment of an efficient surveillance system in several countries such as Japan, where the majority of patients with HCC are diagnosed at an early stage^4^.

The coronavirus disease 2019 (COVID-19) pandemic has had profound and far-reaching effects on global healthcare systems^5^. The COVID-19 pandemic has decreased the number of diagnoses^6,7^, delayed the initiation of treatment^6^, and altered the treatment strategy for cancers^8^. Regarding liver disorders, the diagnosis and antiviral treatment of hepatitis C infection also decreased during the COVID-19 pandemic^9^. Adejumo et al*.* reported the impact of COVID-19 on the treatment of patients with cirrhosis in a large-scale veteran cohort, where the percentage of patients diagnosed by surveillance and the number of newly diagnosed HCC patients decreased during the pandemic^10^. However, Murai et al*.* conducted a multicenter observational study in Japan, and presented that tumor progression at diagnosis and the associated treatment selection for HCC during the COVID-19 pandemic were not changed compared with the era before COVID-19^11^.

We developed a novel nationwide registry (REgistry for Advanced Liver Diseases, REAL) that has stored data for every admission of patients with PLC and decompensated cirrhosis (DC) since 2018^12^. The REAL contains detailed information on the initial and recurrent treatments for PLC and DC from nationwide over 200 institutions. To confirm the real-world impact of the COVID-19 pandemic on HCC clinical practice, we analyzed the status and treatment of HCC before and during the COVID-19 era in Japan.

## Methods

### Study design and participants

In this study, we retrieved data of patients initially diagnosed with HCC between January 2018 and December 2021 from the REAL database^12^. The collected data included anthropometric parameters, viral hepatitis parameters, antiviral treatment history before each admission, hepatic encephalopathy status, ascites, esophageal and gastric varices, tumor characteristics, treatment modalities for PLC and DC, and laboratory data (total bilirubin, serum albumin, serum creatinine, platelet count, and prothrombin time)^12^. We intended to enroll as many patients as possible; only those with missing information on the initial treatment were excluded.

The study was conducted in accordance with the principles of the Declaration of Helsinki. This study complied with the ethical guidelines for medical and health research involving human subjects established by the Japanese Ministry of Education, Culture, Sports, Science, and Technology and the Ministry of Health, Labour, and Welfare. The study protocol was approved by the Research Ethics Committee of the Faculty of Medicine at the University of Tokyo (approval number: 2018053NI). The requirement for individual informed consent was waived by the Research Ethics Committee of the Faculty of Medicine at the University of Tokyo due to the retrospective design of the study. All personal information was anonymized at data entry and an individual identifier was created with a hash function using the patients’ names and birth dates. The study was registered in the University Hospital Medical Information Network Clinical Trial Registry (UMIN000035000). All authors had access to the study data and reviewed and approved the final manuscript.

### Classification of etiology

We classified the patients into four etiologies: hepatitis B virus (HBV), hepatitis C virus (HCV), coinfection with HBV and HCV, and non-B, non-C^12^. Patients were classified as HBV-positive if they were positive for HBs antigen at least once at initial diagnosis or at any admission. Furthermore, patients were classified as having HBV infection if they had a history of receiving antiviral therapy for HBV. Patients were classified as having HCV if they had a positive HCV antibody result at initial diagnosis or at any admission. Furthermore, patients were classified as having HCV infection if they had a history of receiving antiviral therapy. Patients coinfected with HBV and HCV met the criteria for both HBV and HCV infections. The remaining patients were classified as non-B, non-C^12^.

### Diagnosis of primary liver cancer and decompensated cirrhosis

PLC were classified based on the World Health Organization (WHO) classification of Tumours of the Digestive System^13^. HCC was diagnosed pathologically or using imaging criteria based on the Japanese Clinical Practice Guidelines^14^. Hyperattenuation during the arterial phase with washout during the late phase on dynamic computed tomography or dynamic magnetic resonance imaging images was considered a specific finding.

### Definition of before and during the COVID-19 era

COVID-19 was first documented in December 2019^15^ and the WHO declared the COVID-19 outbreak as a global pandemic on March 11, 2020. The study period was divided into the pre-COVID-19 era (January 2018 to December 2019) and the COVID-19 era (January 2020 to December 2021).

### Study outcomes and variables

Among the various indicators of tumor characteristics, we selected tumor size, the most robust and reliable indicator of tumor growth, as the primary outcome measure. We compared the diameter of the maximal intrahepatic lesion at initial diagnosis before and during the COVID-19 era. The following variables were also assessed: number of intrahepatic tumors, vascular invasion, extrahepatic spread, tumor rupture, Barcelona Clinic Liver Cancer (BCLC) stage^16^, duration from diagnosis to initial treatment for HCC, etiology, Child–Pugh score^17^, status of hepatic encephalopathy, ascites, esophageal and gastric varices, tumor characteristics, treatment modalities for HCC, anthropometric parameters, and laboratory data (total bilirubin, serum albumin, serum creatinine, platelet count, and prothrombin time). We further conducted a subgroup analysis stratified by etiology, including HBV, HCV, and non-B, non-C.

### Statistical analysis

Data are presented as means and standard deviations (SD) or medians and interquartile ranges (IQR) for quantitative variables and as numbers and percentages for qualitative variables. The body mass index (BMI), Child–Pugh score, and BCLC stage were calculated using the obtained data (Supplementary Fig. S1). The following unrealistic outliers were treated as missing. Height less than 100 cm, weight less than 10 kg, prothrombin activity less than 10%, and diameter of the maximal intrahepatic lesion greater than 30 cm. BMI, Child–Pugh score, and BCLC stage calculated from these values were also treated as missing.

For comparisons between before and during the COVID-19 era, for continuous variables, Welch’s t-test was used to assess the statistical significance. The Cochran–Armitage trend test was used for statistical analyses of the change in Child–Pugh class, number of intrahepatic tumors, BCLC stage, and tumor markers, and The Chi-squared test was used for other categorical variables. In this study, p < 0.05 was considered statistically significant, and all tests were two-tailed. All statistical analyses were performed using the R software version 4.1 and later (R Foundation, Vienna, Austria, http://www.r-project.org/).

## Results

### Patient characteristics

**Figure 1:**
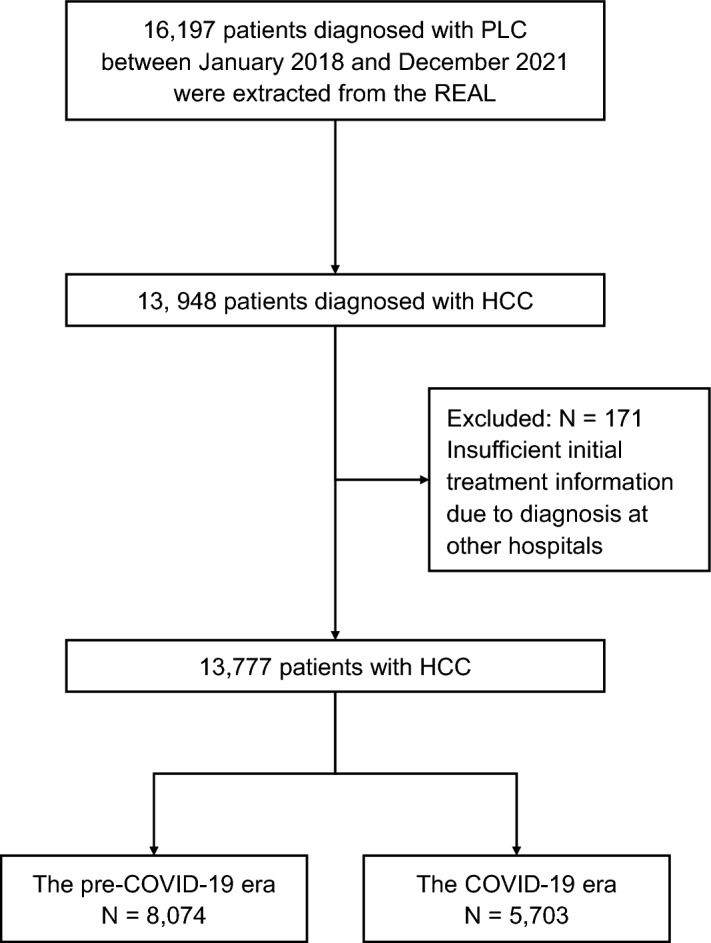
Patient recruitment flowchart. *PLC* primary liver cancer, *HCC* hepatocellular carcinoma, *COVID-19* coronavirus disease 2019.

Data from 16,197 patients initially diagnosed with PLC at 282 hospitals between January 2018 and December 2021 were extracted from the registry. Among them, 13,948 were diagnosed with HCC. One hundred seventy-one patients were excluded because of missing data on initial treatment. The remaining 13,777 patients (8074 in the pre-COVID-19 era and 5703 in the COVID-19 era) were analyzed (Fig. 1). Compared with the pre-COVID-19 era, patients with HCC were older, had a higher BMI, had less viral hepatitis, and more non-B non-C liver diseases in the COVID-19 era (Table 1). These results are consistent with those in our previous report^18^.

**Table 1:** Background characteristics of enrolled patients before and during the era of COVID-19.

Characteristics	Total (n = 13,777)	The pre-COVID-19 era (n = 8074)	The COVID-19 era (n = 5703)	p-value
Background information				
Age, years^a^	72.6 (9.8)	72.4 (9.7)	72.8 (9.9)	0.02
Male gender	10,224 (74.2)	5969 (73.9)	4255 (74.6)	0.37
BMI, kg/m^2b^	24.0 (4.0)	24.0 (4.0)	24.2 (4.1)	< 0.001
Etiology^c^				< 0.001
HBV	1487 (11.2)	921 (11.8)	566 (10.2)	
HCV	4077 (30.6)	2485 (31.9)	1592 (28.8)	
Coinfection with HBV and HCV	109 (0.8)	74 (1.0)	35 (0.6)	
Non-B, non-C	7648 (57.4)	4309 (55.3)	3339 (60.4)	
Child–Pugh class^d^				0.71
A	10,645 (80.7)	6211 (80.7)	4434 (80.6)	
B	2172 (16.5)	1270 (16.5)	902 (16.4)	
C	382 (2.9)	216 (2.8)	166 (3.0)	
Hepatic encephalopathy^e^	292 (2.1)	175 (2.2)	117 (2.1)	0.59
Ascites^f^	1479 (10.8)	845 (10.6)	634 (11.2)	0.28
Esophageal and gastric varices^g^	2104 (18.7)	1280 (19.6)	824 (17.4)	0.004
Total bilirubin, mg/dL^h^	1.1 (1.3)	1.0 (1.2)	1.1 (1.5)	0.06
Albumin, g/dL^i^	3.8 (0.6)	3.8 (0.6)	3.8 (0.6)	0.004
Prothrombin activity, %^j^	88.4 (20.0)	87.9 (19.8)	89.0 (20.3)	0.002
Prothrombin time (INR)^k^	1.1 (0.2)	1.1 (0.2)	1.1 (0.3)	0.72
Platelet, × 10^4^/mm^3l^	18.5 (16.6)	18.2 (16.2)	18.9 (17.0)	0.01
Creatinine, mg/dL^m^	1.0 (1.0)	1.0 (1.1)	1.0 (1.0)	0.78
Tumor characteristics				0.36
Diameter of the maximal intrahepatic lesion, cm^n^	4.4 (3.6)	4.3 (3.6)	4.4 (3.6)	
Number of intrahepatic tumors^o^				0.01
Single	9208 (73.0)	5315 (72.0)	3893 (74.3)	
2–3	2766 (21.9)	1674 (22.7)	1092 (20.8)	
> 3	648 (5.1)	390 (5.3)	258 (4.9)	
Portal vein invasion by imaging^p^	1585 (12.2)	911 (12.0)	674 (12.4)	0.51
Hepatic vein invasion by imaging^q^	775 (6.0)	441 (5.9)	334 (6.2)	0.49
Bile duct invasion by imaging^r^	450 (3.5)	273 (3.6)	177 (3.3)	0.28
Tumor rupture^s^	438 (3.2)	245 (3.1)	193 (3.4)	0.33
Extra hepatic spread^t^	668 (5.0)	378 (4.8)	290 (5.2)	0.38
Time from diagnosis to initial treatment, days^u^	36.5 (30.7)	36.7 (30.9)	36.3 (30.5)	0.45
BCLC stage^v^				0.41
0	2393 (18.4)	1401 (18.5)	992 (18.3)	
A	6547 (50.4)	3798 (50.2)	2749 (50.7)	
B	1654 (12.7)	1013 (13.4)	641 (11.8)	
C	2010 (15.5)	1135 (15.0)	875 (16.1)	
D	382 (2.9)	216 (2.9)	166 (3.1)	
AFP (ng/mL)^w^				0.006
≤ 20	8440 (64.7)	4854 (63.6)	3586 (66.4)	
21–200	2123 (16.3)	1298 (17.0)	825 (15.3)	
> 200	2476 (19.0)	1484 (19.4)	992 (18.4)	
DCP (mAU/mL)^x^				0.04
≤ 100	6498 (50.0)	3865 (50.9)	2633 (48.9)	
101–400	2117 (16.3)	1213 (16.0)	904 (16.8)	
> 400	4369 (33.6)	2518 (33.1)	1851 (34.4)	

Data are expressed as numbers (percentages) or means (standard deviations), unless otherwise indicated.

Data were missing for ^a^11, ^b^221, ^c^456, ^d^578, ^e^127, ^f^134, ^g^2505, ^h^52, ^i^70, ^j^378, ^k^353, ^l^50, ^m^70, ^n^393, ^o^1155, ^p^771, ^q^852, ^r^914, ^s^256, ^t^303, ^u^1053, ^v^791, ^w^738, and ^×^793 patients.

### COVID-19 impact on the patient characteristics and treatment modalities

**Figure 2:**
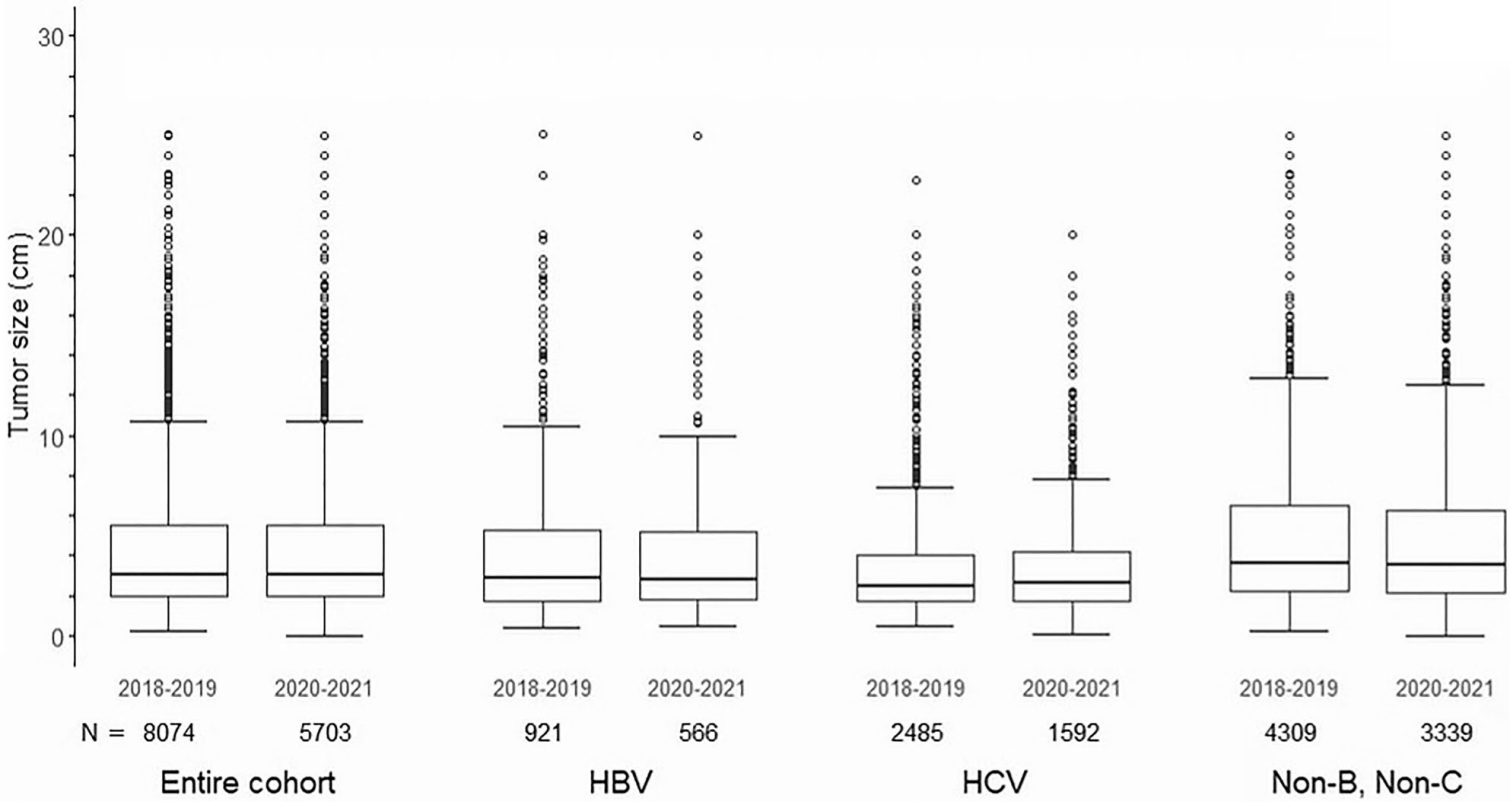
The tumor size in the pre-COVID-19 era and the COVID-19 era stratified by etiologies. The bars represent the means of tumor size of the maximal intrahepatic lesion with standard deviations in each group.

The diameter of the maximal intrahepatic lesion did not differ between the COVID-19 era and before (mean [SD]: 4.4 [3.6] cm vs. 4.3 [3.6] cm; Fig. 2), whereas the proportion of patients with a single tumor increased slightly (72.0% vs. 74.3%) (Table 1). The vascular invasion and extrahepatic spread rates remained unchanged. Liver function, as expressed by Child–Pugh class, was also unchanged. Overall, HCC was diagnosed at similar BCLC stages (0, A, B, C, and D in 18.5% and 18.3%; 50.2% and 50.7%; 13.4% and 11.8%; 15.0% and 16.1%; and 2.9% and 3.1%, respectively). The treatment choice was similar between the two periods, except that the use of systemic therapy increased from 5.4 to 8.9% (Table 2).

**Table 2:** Treatment selection for initially diagnosed HCC before and during the era of COVID-19.

Treatment modality	Total (n = 13,777)	The pre-COVID-19 era (n = 8074)	The COVID-19 era (n = 5703)
Resection^a^	6464 (47.3)	3710 (46.3)	2754 (48.7)
Open	4020 (29.4)	2392 (29.8)	1628 (28.8)
Laparoscopic‐assisted	386 (2.8)	238 (3.0)	148 (2.6)
Laparoscopic	2058 (15.1)	1080 (13.5)	978 (17.3)
Liver transplantation^b^	18 (0.1)	13 (0.2)	5 (0.1)
Ablation^c^	2658 (19.5)	1595 (20.0)	1063 (18.8)
RFA	2320 (17.0)	1424 (17.9)	896 (15.9)
Ethanol injection	34 (0.2)	22 (0.3)	12 (0.2)
Microwave ablation	281 (2.1)	134 (1.7)	147 (2.6)
Others	23 (0.2)	15 (0.2)	8 (0.1)
Transarterial chemoembolization^d^	3967 (29.1)	2479 (31.1)	1488 (26.4)
Radiation^e^	246 (1.8)	174 (2.2)	72 (1.3)
Hepatic arterial infusion chemotherapy^f^	297 (2.2)	202 (2.5)	95 (1.7)
Systemic therapy^g^	930 (6.8)	428 (5.4)	502 (8.9)
Others^h^	63 (0.5)	41 (0.5)	22 (0.4)
Best supportive care	760 (5.5)	439 (5.4)	321 (5.6)

Data were expressed as numbers (percentages).

Data are missing for ^a^110, ^b^110, ^c^165, ^d^166, ^e^204, ^f^166, ^g^185, and ^h^204 patients.

### Subgroup analysis according to the etiology

As the etiology of HCC is rapidly changing in Japan due to the decreased number of patients with chronic hepatitis C^18^, we further analyzed the impact of COVID-19 on patient characteristics stratified by etiology (Table 3). Although the maximum size of the intrahepatic tumors was significantly different among the etiologies, it did not change before and during the COVID-19 era among patients with the same etiology (Fig. 2). Other tumor factors also did not change, except for the increased proportion of single tumors (72.3–75.4%) and portal vein invasion (9.2–12.0%) in the HCV group. Although HCC was diagnosed at similar BCLC stages in each etiology, BCLC C stage was increased in the HCV group. Regarding treatment modalities, the use of systemic therapy also increased in the COVID-19 era for all etiologies (Table 4).

**Table 3:** Background characteristics of initially diagnosed HCC before and in the era of COVID-19 classified with etiology.

Characteristics	HBV (n = 1487)			HCV (n = 4077)			non-B, non-C (n = 7648)		
	2018–2019 (n = 921)	2020–2021 (n = 566)	p-value	2018–2019 (n = 2485)	2020–2021 (n = 1592)	p-value	2018–2019 (n = 4309)	2020–2021 (n = 3339)	p-value
Background information									
Age, years^a^	64.9 (11.2)	65.6 (10.8)	0.23	73.8 (9.0)	73.7 (9.3)	0.67	73.1 (9.1)	73.5 (9.6)	0.09
Male gender	724 (78.6)	444 (78.4)	0.94	1599 (64.3)	1064 (66.8)	0.10	3389 (78.6)	2601 (77.9)	0.43
BMI, kg/m^2b^	23.3 (3.7)	23.8 (3.7)	0.009	23.2 (3.8)	23.3 (3.9)	0.24	24.5 (4.0)	24.6 (4.1)	0.21
Child–Pugh class^c^			0.29			0.88			0.62
A	732 (82.7)	463 (85.4)		1970 (82.9)	1287 (83.2)		3296 (79.5)	2553 (79.0)	
B	129 (14.6)	64 (11.8)		349 (14.7)	215 (13.9)		726 (17.5)	580 (17.9)	
C	24 (2.7)	15 (2.8)		57 (2.4)	45 (2.9)		124 (3.0)	99 (3.1)	
Hepatic encephalopathy^d^	14 (1.5)	9 (1.6)	0.94	52 (2.1)	25 (1.6)	0.22	100 (2.3)	80 (2.4)	0.85
Ascites^e^	82 (9.0)	46 (8.2)	0.59	262 (10.7)	164 (10.3)	0.74	454 (10.7)	399 (12.0)	0.06
Esophageal and gastric varices^f^	127 (16.8)	65 (13.8)	0.15	425 (21.4)	240 (18.5)	0.047	664 (18.8)	479 (17.1)	0.09
Total bilirubin, mg/dL^g^	1.0 (0.9)	1.3 (2.5)	0.01	1.0 (0.8)	1.1 (1.5)	0.02	1.1 (1.4)	1.1 (1.2)	0.28
Albumin, g/dL^h^	3.9 (0.6)	4.0 (0.6)	0.16	3.9 (0.6)	3.9 (0.6)	0.01	3.8 (0.6)	3.8 (0.6)	0.03
Prothrombin activity, % ^i^	88.6 (20.6)	89.9 (20.0)	0.23	89.2 (18.6)	90.0 (18.7)	0.17	87.4 (20.2)	88.8 (20.8)	0.006
Prothrombin time (INR)^j^	1.1 (0.2)	1.1 (0.2)	0.86	1.1 (0.3)	1.1 (0.3)	0.95	1.1 (0.2)	1.1 (0.3)	0.61
Platelet, × 10^4^/mm^3k^	18.2 (14.9)	19.6 (14.5)	0.07	16.8 (16.3)	17.3 (15.4)	0.30	18.9 (15.7)	19.6 (17.8)	0.06
Creatinine, mg/dL^l^	0.9 (1.2)	0.9 (0.7)	0.38	1.0 (1.0)	1.0 (1.1)	0.23	1.0 (1.1)	1.0 (1.0)	0.50
Tumor characteristics									
Diameter of the maximal intrahepatic lesion, cm^m^	4.2 (3.8)	4.3 (3.8)	0.89	3.5 (3.0)	3.6 (2.9)	0.67	4.9 (3.7)	4.8 (3.8)	0.46
Number of intrahepatic tumors^n^			0.49			0.04			0.14
Single	640 (76.2)	388 (77.4)		1680 (72.3)	1123 (75.4)		2785 (71.2)	2243 (73.1)	
2–3	163 (19.4)	95 (19.0)		534 (23.0)	305 (20.5)		906 (23.1)	653 (21.3)	
> 3	37 (4.4)	18 (3.6)		111 (4.8)	61 (4.1)		223 (5.7)	172 (5.6)	
Portal vein invasion by imaging^o^	113 (13.0)	71 (13.1)	0.98	216 (9.2)	183 (12.0)	0.005	557 (13.7)	400 (12.6)	0.15
Hepatic vein invasion by imaging^p^	54 (6.3)	35 (6.5)	0.90	107 (4.6)	79 (5.2)	0.39	267 (6.6)	212 (6.7)	0.94
Bile duct invasion by imaging^q^	31 (3.6)	26 (4.8)	0.29	53 (2.3)	27 (1.8)	0.30	179 (4.5)	121 (3.8)	0.20
Tumor rupture^r^	31 (3.5)	20 (3.6)	0.92	58 (2.4)	51 (3.2)	0.12	147 (3.5)	117 (3.5)	0.87
Extra hepatic spread^s^	45 (5.1)	30 (5.3)	0.81	89 (3.7)	53 (3.4)	0.59	234 (5.5)	198 (6.0)	0.40
Time from diagnosis to initial treatment, days^t^	35.4 (29.9)	38.5 (32.5)	0.08	36.3 (30.4)	36.6 (30.9)	0.79	37.4 (31.4)	35.7 (29.9)	0.02
BCLC stage^u^			0.53			0.19			0.69
0	218 (25.0)	148 (27.8)		605 (25.8)	385 (25.2)		512 (12.6)	424 (13.3)	
A	399 (45.7)	232 (43.6)		1119 (47.8)	734 (48.0)		2158 (52.9)	1689 (52.9)	
B	88 (10.1)	51 (9.6)		287 (12.3)	149 (9.8)		596 (14.6)	430 (13.5)	
C	144 (16.5)	86 (16.2)		273 (11.7)	215 (14.1)		688 (16.9)	550 (17.2)	
D	24 (2.7)	15 (2.8)		57 (2.4)	45 (2.9)		124 (3.0)	99 (3.1)	
AFP (ng/mL)^v^			0.09			0.19			0.10
≤ 20	517 (58.9)	340 (63.2)		1482 (62.2)	980 (64.5)		2671 (65.3)	2154 (67.9)	
21–200	156 (17.8)	90 (16.7)		460 (19.3)	275 (18.1)		638 (15.6)	428 (13.5)	
> 200	205 (23.3)	108 (20.1)		441 (18.5)	265 (17.4)		784 (19.2)	592 (18.7)	
DCP (mAU/mL)^w^			0.02			0.03			0.30
≤ 100	501 (56.2)	267 (49.0)		1408 (59.8)	833 (55.3)		1795 (44.0)	1439 (45.5)	
101–400	127 (14.2)	92 (16.9)		337 (14.3)	259 (17.2)		712 (17.5)	531 (16.8)	
> 400	264 (29.6)	186 (34.1)		611 (25.9)	415 (27.5)		1569 (38.5)	1193 (37.7)	

Data are expressed as numbers (percentages) or means (standard deviations), unless otherwise indicated.

Data were missing for ^a^11, ^b^221, ^c^578, ^d^127, ^e^134, ^f^2505, ^g^52, ^h^70, ^i^378, ^j^353, ^k^50, ^l^70, ^m^393, ^n^1155, ^o^771, ^p^852, ^q^914, ^r^256, ^s^303, ^t^1053, ^u^791, ^v^738 and ^w^793 patients.

**Table 4:** Treatment selection of initially diagnosed HCC before and in the era of COVID-19 classified with etiology.

Treatment modality	HBV (n = 1487)		HCV (n = 4077)		non-B, non-C (n = 7648)	
	2018–2019 (n = 921)	2020–2021 (n = 566)	2018–2019 (n = 2485)	2020–2021 (n = 1592)	2018–2019 (n = 4309)	2020–2021 (n = 3339)
Resection^a^	507 (55.3)	335 (59.6)	1075 (43.6)	735 (46.5)	2047 (47.8)	1619 (49.0)
Open	321 (35.0)	202 (35.9)	638 (25.9)	396 (25.0)	1372 (32.0)	986 (29.9)
Laparoscopic‐assisted	34 (3.7)	16 (2.8)	84 (3.4)	42 (2.7)	117 (2.7)	87 (2.6)
Laparoscopic	152 (16.6)	117 (20.8)	353 (14.3)	297 (18.8)	558 (13.0)	546 (16.5)
Liver transplantation^b^	6 (0.7)	0 (0.0)	0 (0.0)	1 (0.1)	6 (0.1)	4 (0.1)
Ablation^c^	164 (17.9)	91 (16.2)	655 (26.8)	361 (22.8)	665 (15.6)	552 (16.7)
RFA	152 (16.6)	79 (14.1)	577 (23.6)	305 (19.3)	591 (13.9)	457 (13.8)
Ethanol injection	0 (0.0)	2 (0.4)	8 (0.3)	4 (0.3)	11 (0.3)	6 (0.2)
Microwave ablation	11 (1.2)	8 (1.4)	64 (2.6)	49 (3.1)	57 (1.3)	86 (2.6)
Others	1 (0.1)	2 (0.4)	6 (0.2)	3 (0.2)	6 (0.1)	3 (0.1)
Transarterial chemoembolization^d^	206 (22.6)	115 (20.5)	764 (31.3)	463 (29.3)	1374 (32.2)	838 (25.4)
Radiation^e^	30 (3.3)	10 (1.8)	47 (1.9)	19 (1.2)	96 (2.3)	42 (1.3)
Hepatic arterial infusion chemotherapy^f^	21 (2.3)	9 (1.6)	58 (2.4)	26 (1.6)	116 (2.7)	57 (1.7)
Systemic therapy^g^	57 (6.3)	58 (10.3)	82 (3.4)	105 (6.7)	263 (6.2)	322 (9.8)
Others^h^	4 (0.4)	1 (0.2)	12 (0.5)	4 (0.3)	23 (0.5)	16 (0.5)
Best supportive care	29 (3.1)	17 (3.0)	113 (4.5)	82 (5.2)	251 (5.8)	202 (6.0)

Data were expressed as numbers (percentages).

Data are missing for ^a^110, ^b^110, ^c^165, ^d^166, ^e^204, ^f^166, ^g^185, and ^h^204 patients.

## Discussion

This study clarified the real-world clinical practice of initially diagnosing HCC in Japan before and during the COVID-19 pandemic. Delayed diagnosis manifests in various ways, such as changes in the tumor stage and treatment options. We assessed tumor size, which is the simplest and most reliable indicator of tumor growth. The results showed that the size of the maximal intrahepatic tumor remained unchanged between the two periods, even after stratification by etiology.

The Japanese Practice Guidelines for HCC recommend that patients with HCV, HBV, or cirrhosis of any etiology should be surveillance candidates^14^. The effectiveness of surveillance was indicated by the percentage of candidates who underwent surveillance. Although our database does not have information on whether a patient was diagnosed through surveillance, the results suggest that the vast majority of patients at high risk of HCC development, especially HCV patients in whom the tumor size was the smallest among the etiologies, did not drop out of surveillance during the COVID-19 era.

The proportion of non-viral etiologies of HCC has increased over the past two decades, reflecting a decrease in HCV-associated patients and an increase in lifestyle-related diseases^18^. This trend was also observed in the present study, and it was not possible to determine a causal relationship between the increase of non-B, non-C and the COVID-19 pandemic. The size of the intrahepatic tumors differed significantly between etiologies, namely the order of the sizes was non-B, non-C, HBV, and HCV. Although the proportion of non-B, non-C increased, it was not enough to significantly increase the tumor size and treatment selection in entire cohort. To highlight the difference owing to COVID-19 pandemic, we further analyzed the characteristics and treatments stratified by etiology. The size of intrahepatic tumors of each etiology was also unchanged between the two periods. Other tumor factors were also unchanged, except for an increased proportion of HCV patients with a single nodule. Other findings in patients with HCV infection include a decreased incidence of esophageal and gastric varices and increased serum albumin levels during the COVID-19 era. This is probably due to an increase in the number of patients who achieved a sustained virological response (SVR) with direct-acting antivirals in Japan, where access to these drugs is publicly established for all patients with HCV^19,20^. On the other hand, the proportion of portal vein invasion increased during the COVID-19 era, resulting in an increase in the percentage of patients in BCLC C stage. This paradoxical trend may indicate that some patients dropped out of the surveillance after achieving SVR^21^.

Systemic therapies for HCC have made great strides over the past few years. In particular, combination treatment with atezolizumab and bevacizumab, which became available in September 2020, was superior to sorafenib in a phase III trial for unresectable HCC^22^, resulting in the replacement of first-line therapy with combination immunotherapy from sorafenib or lenvatinib^23^. The major target population for systemic therapy in HCC is BCLC stage C, that is, patients with vascular invasion or extrahepatic metastasis^22,24,25^. Increased number of systemic therapy regimens has contributed to a shift in treatment options for BCLC stage B from TACE to systemic therapy in Japan^26^. The present study demonstrated that more patients were treated with systemic therapy in the COVID-19 era after the introduction of atezolizumab and bevacizumab combination therapy.

Our study had some limitations. First, the majority of participating facilities in the REAL are tertiary care centers where HCC treatments are usually provided. Therefore, the results obtained in this study may not be fully extrapolated to primary or secondary hospitals. Second, we could not assess the direct effect of COVID-19 pandemic on the adherence to surveillance, since the REAL did not collect data on the diagnostic process. However, since the participating hospitals were the same between the pre-COVID-19 and COVID-19 eras, we believe that we could access the comprehensive impact of COVID-19 pandemic on the diagnosis of HCC. Third, the number of patients diagnosed HCC in two years was decreased from 8074 to 5703 in the COVID-19 era. This decline might suggest that there are potentially patients who have not yet been registered to the registry, underreporting, or delayed diagnoses due to the pandemic. The REAL is continuously accumulating HCC cases and future analyses might provide further consideration. Fourth, COVID-19 may have affected various components of the HCC diagnostic process, such as the recruitment of high-risk populations for surveillance, detection of nodules by ultrasonography, and confirmation of HCC by dynamic CT or MRI. Although we found that the tumor size did not change as a composite outcome, we could not separately assess the impact of each component. Nevertheless, we can conclude that the overall situation has not changed during the COVID-19 pandemic. Given the relatively easy access to diagnostic imaging in Japan, a more robust recruitment and surveillance system, particularly for non-viral etiology including metabolic dysfunction-associated steatotic liver disease, should be in place to prepare for future pandemics. Based on the lessons learned from the COVID-19 pandemic, it is also recommended that each guideline should include the surveillance system in advance at the situation of future pandemics.

In conclusion, the treatment status of initially diagnosed HCC generally remained unchanged during the COVID-19 pandemic in Japan, even after accounting for differences in etiology. A robust surveillance system should be established particularly for non-B, non-C etiology to detect HCC in earlier stages.

### Supplementary Information

Supplementary Figure 1.

## Data Availability

The datasets generated during the current study are not publicly available due to further uses for clinical studies in the future but are available from the corresponding author on reasonable request.

## Abbreviations

PLC: Primary liver cancer
HCC: Hepatocellular carcinoma
COVID-19: Coronavirus disease 2019
REAL: Registry for advanced liver diseases
DC: Decompensated cirrhosis
HBV: Hepatitis B virus
HCV: Hepatitis C virus
WHO: World Health Organization
SD: Standard deviation
IQR: Interquartile range
BCLC: Barcelona clinic liver cancer
BMI: Body mass index
SVR: Sustained virological response
