# Supplementary material for: The impact of COVID-19 on the diagnosis and treatment of HCC: analysis of a nationwide registry for advanced liver diseases (REAL)
Source: Sci Rep. 2024 Feb 3;14:2826. doi: 10.1038/s41598-024-53199-6 (PMC10838269; doi:10.1038/s41598-024-53199-6)
Supplement: Supplementary file 1 — Supplementary Figure 1. [file 41598_2024_53199_MOESM1_ESM.pptx]

## Slide 1
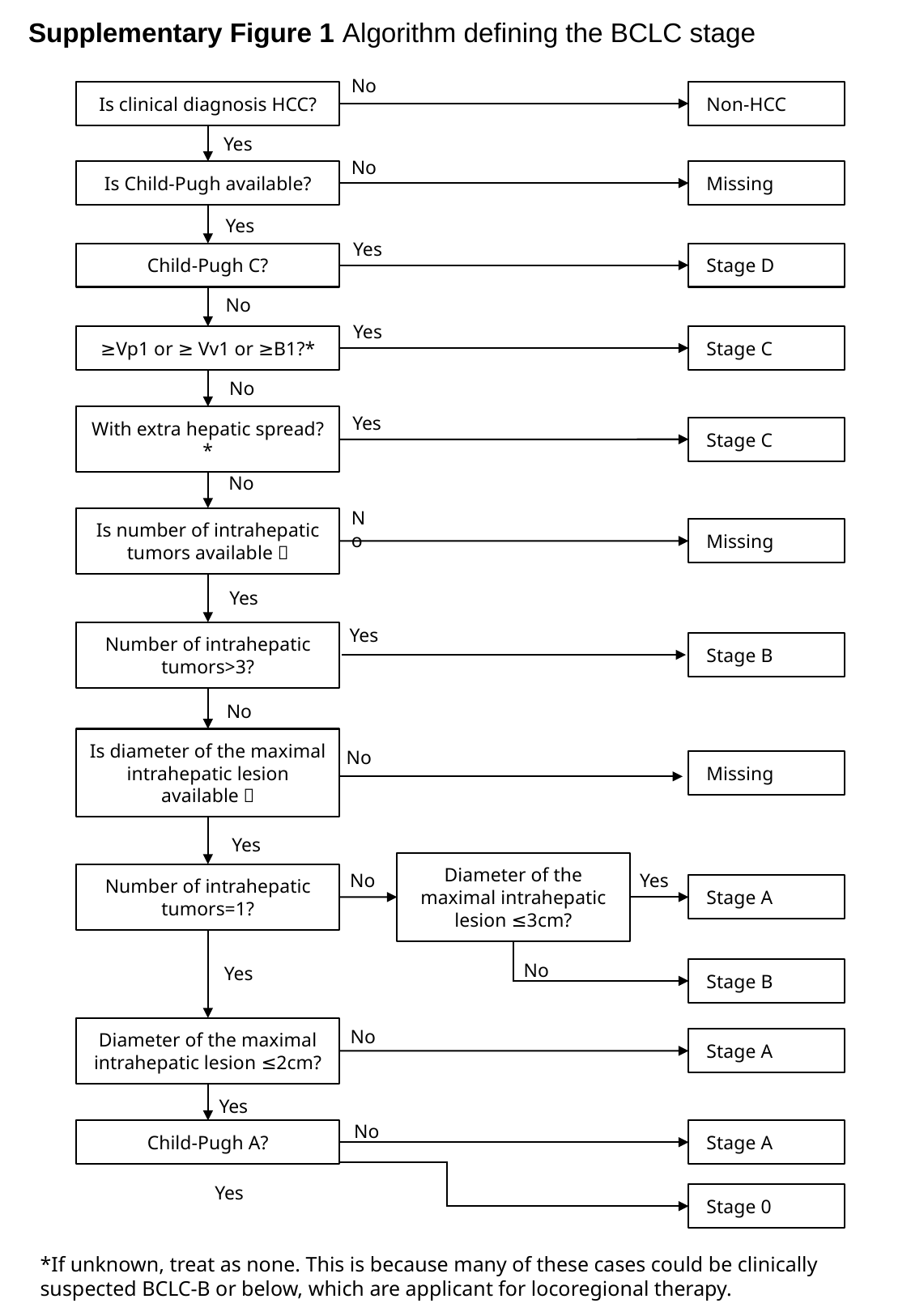

Supplementary Figure 1 Algorithm defining the BCLC stage
No
Is clinical diagnosis HCC?
Non-HCC
Yes
No
Is Child-Pugh available?
Missing
Yes
Yes
Child-Pugh C?
Stage D
No
Yes
≥Vp1 or ≥ Vv1 or ≥B1?*
Stage C
No
Yes
With extra hepatic spread?*
Stage C
No
No
Is number of intrahepatic tumors available？
Missing
Yes
Yes
Number of intrahepatic tumors>3?
Stage B
No
Is diameter of the maximal intrahepatic lesion available？
No
Missing
Yes
Diameter of the maximal intrahepatic lesion ≤3cm?
Yes
No
Number of intrahepatic tumors=1?
Stage A
No
Yes
Stage B
Diameter of the maximal intrahepatic lesion ≤2cm?
No
Stage A
Yes
No
Child-Pugh A?
Stage A
Yes
Stage 0
*If unknown, treat as none. This is because many of these cases could be clinically suspected BCLC-B or below, which are applicant for locoregional therapy.
